# Supplementary material for: Using the Brief Health Literacy Screen in Chronic Care in French Hospital Settings: Content Validity of Patient and Healthcare Professional Reports
Source: Int J Environ Res Public Health. 2020 Dec 25;18(1):96. doi: 10.3390/ijerph18010096 (PMC7795429; doi:10.3390/ijerph18010096)
Supplement: Supplementary file 1 [file ijerph-18-00096-s001.zip › S1_table_Comparison of the 2 BHLS versions and proposition of change in English and in French.docx]

**Table S1.** Comparison of the 2 BHLS versions and proposition of change in English and in French

In bold, the changes made after cognitive interviews with patients and in italics the French version.

|  | **Patient-reported BHLS V1** | **Patient-reported BHLS V2** | **HCP-reported BHLS V1** |
| --- | --- | --- | --- |
| Q1 (English/*French*) | How confident are you filling forms by yourself? / *A quel point êtes- vous confiant(e) lorsque vous complétez des formulaires par vous-mêmes?* | How confident are you filling **medical forms** by yourself? / *A quel point êtes-vous confiant(e) lorsque vous complétez des* ***formulaires médicaux*** *par vous- mêmes ?* | **In your opinion, to what extent the patient is** confident to fill in medical forms by him/herself? / ***Selon vous, à quel point, le patient est -il*** *confiant pour remplir lui-même ses formulaires médicaux?* |
| Q2 (English/*French*) | How often do you have someone help you read hospital materials? / *A quelle fréquence vous arrive-t-il de demander de l’aide pour lire la documentation remise à l’hôpital?* | How often do you have someone help you read (family, friends, neighbors, doctors, pharmacists) hospital materials? / *À quelle fréquence vous arrive – t- il de demander de l’aide* ***(proches: familles, amis, voisins)****pour lire la documentation remise à l’hôpital?* | **In your opinion**, how often does **he/she** have someone (family, friends, neighbors, doctors, pharmacists) help **him/her** read hospital materials? / ***Selon vous****, à quelle fréquence* ***le patient a -t-il*** *besoin de demander de l’aide pour lire la documentation remise à l’hôpital?* |
| Q3 (English/*French*) | How often do you have problems learning about your medical conditions because of difficulty reading hospital materials? / *A quelle fréquence vous arrive-t-il d’éprouver de la difficulté à comprendre votre condition médicale parce que la documentation qui vous est remise est difficile à lire?* | How often do you have problems learning about your **health status** because of hard-to-read materials? / *À quelle fréquence vous arrive –t-il d’éprouver de la difficulté à comprendre votre* ***état de santé à cause d’une documentation difficile à lire****?* | **In your opinion**, how often does **he/she have** problems learning about **his/her** health status because of difficulties reading documents delivered by the hospital?  / ***Selon vous****, à quelle fréquence* ***le patient rencontre-t-il*** *des difficultés à comprendre son état de santé à cause de difficultés à lire les documents qui lui sont remis?* |
