# Supplementary material for: Using the Brief Health Literacy Screen in Chronic Care in French Hospital Settings: Content Validity of Patient and Healthcare Professional Reports
Source: Int J Environ Res Public Health. 2020 Dec 25;18(1):96. doi: 10.3390/ijerph18010096 (PMC7795429; doi:10.3390/ijerph18010096)
Supplement: Supplementary file 1 [file ijerph-18-00096-s001.zip › S2_table_Synthesis of patients cognitive interviews in French.docx]

**Table S2.** Synthesis of patients’ cognitive interviews in French

|  | **Patient 1** | **Patient 2** | **Patient 3** | **Patient 4** | **Patient 5** | **Patient 6** | **Patient 7** |
| --- | --- | --- | --- | --- | --- | --- | --- |
| **Q1** |  |  |  |  |  |  |  |
| **"formulaire"** | Prise en charge  Ordonnance  (Document à remplir ou non) | Formulaires médicaux / de santé  Questionnaire | Formulaire sur la BPCO | Etat de santé  Données utiles pour le médecin | Questionnaire en général | Questionnaires de santé pour compléter l’approche du diagnostic par le médecin | Questionnaire en général |
| **"être confiant"** | Compréhension | Avoir confiance | Se laisser guider par ce que dit le médecin | Relation entre médecin – patient  🡪 Avoir confiance en le médecin | Confiance vis-à-vis du questionnaire et en leurs créateurs  🡪 Avoir confiance ou non dans un élément proposé | Questions ciblant bonnes informations    → Pas de rapport avec la confiance en soi | Etre sûr de soi |
| **Situations référentes** | En général | Hospitalisations  Traitements | Consultation du jour / Formulaire BPCO | Formulaire sur l’asthme | Remplissage de documents confidentiels (« passeport, Carte d’identité» | Questionnaire rempli avant la consultation du jour |  |
| **Effort réalisé** | Réponses spontanées | Réponses spontanées et rapides | Réponses données instinctivement | Réponses données instinctivement | Réponses spontanées | Non | Réponses machinales |
| **Ressenti influencé / orienté** | Non | Non | Non | Non | Non |  | Non |
| **Représentation modalité** | « Très confiant »  🡪possible problème de compréhension mais rare | « Assez confiante »  = avoir confiance  → pas de distinction avec « très confiante » | « Assez confiante »  = pas totalement sûr | «Très confiante » | « Assez confiant »  = confiance en le médecin | « Assez confiant »  = confiance mutuelle médecin – patient | « Très confiant »  = 100% |
| **Q2** |  |  |  |  |  |  |  |
| **"Demander de l'aide"** | NK  Dépendant de difficulté et compréhension du document | Secrétaire  Médecin | Médecin  Jamais les proches | Aide – soignante    Infirmière | Médecin | NK  Dépend du type de document  → personne concernée par le secteur de la documentation (médecin, administration) | Proches , personnel médical |
| **"Documentation"** | Ordonnances  Termes médicaux  (écrit) | Papier informant les gens sur traitement  Ordonnances  (écrit) | Ordonnances  Résultats d’analyses  (écrit) | Prospectus disponibles en salle d’attente  (écrit) | Documents des traitements, de la pathologie  (écrits) | Tous types de documents : plaquettes d’infos (sur pathologie, alimentation), questionnaire de sortie  → Pas référence à ordonnance | Brochures (écrit) |
| **Situations référentes** |  | Documentation (piqûre l’infirmière) | Problèmes aigus  Consultation  Examens à passer | Salle d’attente à l’hôpital  Consultation médicale | Résultats d’analyses |  | Rares visites à l’hôpital |
| **Représentation modalité** | « Occasionnellement »  = 1 fois de temps en temps | « Jamais »  → pose des questions sur ordonnance/ traitement | « Jamais » = zéro  → demande si ne connais pas | « Jamais » = zéro | « Occasionnellement » = peu souvent  → demande si ne comprend pas | « Occasionnellement » = a priori pas besoin mais l’occasion peut se présenter | « Jamais »  = très rare |
| **Q3** |  |  |  |  |  |  |  |
| **"difficulté à comprendre"** |  |  |  |  | Pour comprendre les termes employés | Documentation pas assez explicative  Documentation non suffisante pour comprendre | Compréhension lors de la 1ère lecture |
| **"condition médicale"** | Condition/ situation actuelle : avoir une difficulté de compréhension - situation dans laquelle on est | Traitements  Soins | Diagnostic    État de santé | État de santé  Maladie (asthme) | Par rapport à sa maladie et explications du médecin | Prise en charge au sens global :  (Soins, secrétariat, accueil administratif) | « Bien être » médical |
| **"difficile à lire"** | Termes techniques (ordonnances)  Lisibilité (écriture médecin) | Papier clair  (Termes trop techniques)  Lisibilité : difficile à déchiffrer (écriture médecin) |  |  |  |  | Termes techniques non compris |
| **Situations référentes** | Informations compliquées à saisir ou pas assez expliquées | écriture des médecins (« difficile à lire » ) |  | Propre maladie | Consultation pour sa situation personnelle |  |  |
| **Représentation modalité** | « Souvent » | « Jamais »  → pose des questions sur ordonnance | « Jamais » | « Jamais »  → n’hésite pas à poser des questions | « Occasionnellement » = pas souvent | « Occasionnellement » | « Jamais » |
| **Sujet abordé selon le patient** | Améliorer les termes pour une compréhension plus simple et plus rapide sur les questionnaires ou autres documents | Comprendre ses formulaires et documentations | Visite avec le médecin |  |  |  | Bien être du patient en général |
| **Difficultés des questions** | Oui pour certaines personnes | Assez simple | Assez facile | 3ème question compliquée |  | Pas de difficultés particulières |  |
| **Remarques** | Questionnaire clair  Modifier « Condition médicale» | Préciser « formulaire »  → formulaire médical | Non | Regrouper les modalités  🡪 Très confiant – Un peu confiant –Pas du tout confiant | Questionnaire compréhensible et lisible | Terme « littératie » non connu | Revoir le titre : « Questionnaire de santé »  Termes assez vague : documents, demander de l’aide |

Continuation of the table S2

|  | **Patient 8** | **Patient 9** | **Patient 10** | **Patient 11** | **Patient 12** | **Patient 13** |
| --- | --- | --- | --- | --- | --- | --- |
| **Q1** |  |  |  |  |  |  |
| **"formulaire"** | Formulaires administratifs (nom, prénom ..) | Document à remplir en général | Questionnaire | Documents explicatifs  Papiers importants dans le cadre général | Questionnaires de satisfaction remis aux patients | Questionnaires à remplir  Dans le cadre général |
| **"être confiant"** | Hésiter pour répondre ou non  Comprendre ce que l’on me demande | Sûr et convaincu de ce que l’on remplit | Être juste dans les réponses fournies | Avoir confiance en soi | Être sûr de soi dans la compréhension des questions | Confiance en ceux à qui on s’adresse (destinataires questionnaires) |
| **Situations référentes** | Consultation du jour : Compléter formulaire sur l’asthme | Vécu = expérience personnelle |  | État de santé : propre expérience, rendez-vous | Propre profession | Remplir formulaires du quotidien Ex : impôts |
| **Effort réalisé** | Non | Non Réponses venues rapidement / instinctivement | Non | Non | Non | NA |
| **Ressenti influencé / orienté** | Non | Non | Non | Non | Non | NA |
| **Représentation modalité** | « Très confiante »  = aucune difficulté | « Très confiant »  = ne se pose pas de question (90%) | « Très confiant »  = persuadé de bien répondre, sûr, sans faute | « Assez confiante » = en général confiante mais parfois un doute | « Très confiant » = spontané, répondre rapidement | « Assez confiant » |
| **Q2** |  |  |  |  |  |  |
| **"Demander de l'aide"** | Infirmière, médecin, secrétaire | Personne maitrisant mieux le sujet : Professionnel de santé | Médecin remettant documentation  Professionnel de santé uniquement  → opposé à dialogue | Médecins, infirmières, aides-soignantes, spécialistes | Infirmière, aide-soignante | Souvent les proches (famille) |
| **"Documentation"** | Documents administratifs  Résultats d’examen | Livret d’accueil  Résultats d’examens | Informations sur soins, traitements, médicaments  (Ecrit et oral) | Informations sur origines et caractéristique de la maladie  Infos orales | Questionnaire de satisfaction /  Documentation sur des pathologies - acte médical – anesthésie..  (écrit) |  |
| **Situations référentes** |  | Contact avec médecin en unité de soins |  |  | Propre profession |  |
| **Représentation modalité** | « Jamais »  = zéro | « Jamais »  = pas du tout  → demande de l’aide au médecin  → Pas demander de l’aide mais expliquer infos | « Jamais »  = zéro | « Jamais » = zéro | « Jamais » = zéro | « Jamais » = quelques fois |
| **Q3** |  |  |  |  |  |  |
| "difficulté à comprendre" | Relatifs aux termes médicaux, scientifiques |  | Langage des médecins |  | Avoir un doute sur expression – phrase- formulation | Termes employés |
| "condition médicale" | Pathologie  Etat de santé | Dossier médical  Pathologies | Actualité médicale | Dossier médical | Etat de patient | Conditions données par le médecin |
| **"difficile à lire"** | Mots inconnus donnés par le médecin | Termes médicaux complexes | Termes spécifiques à des services | Lecture difficile des résultats d’examens | Langage très médicalisé | Termes employés |
| **Situations référentes** | Rendez vous avec son médecin, a compris tout ce qui lui disait | Propre pathologie | Hospitalisation passée et récente |  |  | Ne comprend pas toujours les termes → se fait expliquer |
| **Représentation modalité** | « Jamais » | « Jamais » | « Occasionnellement »  = difficulté à comprendre lors de la première lecture | « Parfois » = de temps en temps | « Jamais » | « Parfois » |
| **Sujet abordé selon le patient** |  | Hospitalisation et état de santé |  |  | Compréhension des documents |  |
| **Difficulté des questions** | Questions ambiguës pour certaines personnes | Simples | Simples | Difficulté avec la troisième question | Simples sauf la troisième question |  |
| **Remarques** | Revoir « condition médicale »  et « documentation » car interprétation peut différer selon le contexte |  | « Littératie » = non adapté car non compris par les patients (comme les termes complexes des médecins) |  | Troisième question trop longue |  |

NA: Non application, NK: Not Known, Ex: Example, BPCO: Bronchopneumopathie chronique obstructive or chronic obstructive pulmonary disease
