# Supplementary material for: Using the Brief Health Literacy Screen in Chronic Care in French Hospital Settings: Content Validity of Patient and Healthcare Professional Reports
Source: Int J Environ Res Public Health. 2020 Dec 25;18(1):96. doi: 10.3390/ijerph18010096 (PMC7795429; doi:10.3390/ijerph18010096)
Supplement: Supplementary file 1 [file ijerph-18-00096-s001.zip › S3_table_Synthesis of HCPs cognitive interviews in French.docx]

**Table S3.** Synthesis of HCPs’ cognitive interviews in French

|  | **PS 1** | **PS 2** | **PS 3** | **PS 4** | **PS 5** | **PS 6** | **PS 7** |
| --- | --- | --- | --- | --- | --- | --- | --- |
| **Q1** |  |  |  |  |  |  |  |
| **« être confiant »** | Confiance en soi | Confiance en soi / confiance en le système de sante / confiance en les professionnels de santé | Confiance en soi Ex : « être confiant, c’est penser pouvoir le faire sans erreur » | Confiance en soi Ex : « est-ce qu’il se sent confiant dans le sens 'sûr de lui' ? » | Confiance en soi | Confiance en soi  Ex : “sûr de lui” | Confiance en soi / confiance en les professionnels de santé |
| **« formulaire médical »** | OUI  Ex: "ordonnance", "un consentement pour un examen médical, une prise de sang” | OUI  Ex: “Consentement”, “personne de confiance” , “arrêt de travail” , “formulaire sortie contre avis médical” | OUI | OUI  Ex: «questionnaire dans les études » | OUI | OUI  Ex: “autorisation d’opérer”, “ordonnance” | OUI  Ex: “prescriptions”, “renseignements d'antécédents” , “documents pour désigner une personne de confiance” |
| **Situations référentes** | OUI  Ex : « j’ai pensé à un patient bah hier tient qui est rentré pour un bilan d’hypertension (...) Je lui ai passé le consentement" | NON | NON Pratique en générale | OUI | NON | NON  Ex : “je l’ai imaginé car je me souviens des situations” | NON |
| **Représentation modalité** | Moyennement confiant | Assez confiant Ex : « me semble bien éduquée, bien investie dans sa maladie. A déjà réfléchis à tous les formulaires qu’on lui a donné" | Assez confiant Ex : « disons qu’il était assez autonome pour pouvoir les remplir et penser mettre des réponses orientées et judicieuses » | Un peu confiant Ex : « un patient avec un certain degré d’incertitude car pas encore très bien et pas suffisamment informé sur la maladie » | Moyennement confiant Ex : "aurais pu dire ce qu'il avait mais avec des oublis" | Assez confiant Ex : “difficile car on se met à la place de l’autre, c’est mon ressenti, je fais que d’interpréter” “je lui fais confiance car il est patient mais pas moins médecin” | Moyennement confiant Ex : « je me suis pas trop engagé j’ai mis entre les deux" |
| **Q2** |  |  |  |  |  |  |  |
| **« demander de l'aide »** | Proche / sur la documentation / questions orales | Professionnel de santé / appel téléphonique / sur les soins | Professionnel de santé / questions orales / sur la documentation | Proches / questions orales | Proches/ questions orales | NA | Proches / sur la documentation |
| **« documentation remise à l'hôpital »** | Documents généraux, documents/spécifiques  Ex : « livret d’accueil, une présentation du service, le questionnaire de sortie et le désignation d’une personne de confiance »  Ex :« les inversions alimentaires… fait partie de la check-list de l’entrée du patient » | Documents généraux /documents spécifiques  Ex : « courrier d’hospitalisation, ordonnance, prochains rdv : à la sortie » "petits prospectus durant l’hospitalisation" | Documents spécifiques (informatif/éducatif sur sa maladie) Ex : « documentation d’information au visé du patient » | Documents généraux /documents spécifiques | Documents spécifiques (relatif a la maladie, aux soins)  Ex : "prise de rendez vous", "prescription", "contre rendu | Documents spécifiques (informatif/éducatif sur sa maladie, relatif à la maladie, aux soins) | Documents généraux /documents spécifiques |
| **Situations référentes** | Oui hospitalisation | Non | Oui hospitalisation | Non | Oui consultation | Non | Non |
| **Représentation modalité** | Jamais | Occasionnellement | Occasionnellement Ex : « à chaque fois qu’on le voyait il avait toujours des questions complémentaires à poser » | Parfois | Souvent Ex : “peut être il va essayer de le déchiffrer lui même mais il va le passer systématiquement à quelqu’un d’autre” | Jamais Ex : “je lui fais toujours confiance, intellectuellement il a la capacité de comprendre il a fait médecine.” | Parfois |
| **Q3 BHLS** |  |  |  |  |  |  |  |
| **«difficultés à comprendre »** | Complexité de la maladie, complexité des documents | Complexité des documents | Littératie générale « quand on remet un document au patient, est-ce qu’il comprend ne comprend pas parce qu’il n’arrive pas à lire les mots qui sont dessus » | Complexité des termes | Complexité des documents | Complexité des termes | Complexité de la maladie, littératie générale |
| **« état de santé »** | Conclusion de la prise en soins | Conclusion de la prise en soins | Maladie en elle-même | NA | Maladie en elle-même, conclusion de la prise en soins | Maladie en elle-même, conclusion de la prise en soins “est-ce qu’il comprend les causes et ces causes vont induire des difficultés au niveau de la santé” | Conclusion de la prise en soins |
| **"difficultés à lire"** | Littératie générale, complexité des termes | Complexité des termes | Littératie générale Ex : « c’est relatif aux difficultés à vraiment lire la langue française et les documents » | Littératie générale | Complexité des termes | Littératie générale, complexité des termes | Complexité des termes |
| **Situations référentes** | NON Ex : « Je pensais plus à des patients… pas la personne dont je vous ai parlé d’hier mais d’autres patients qui reviennent régulièrement" | OUI | NON | NON Ex :"Beaucoup de migrant ou en situation très traumatique (situation irrégulières qui parlent pas la langue) peuvent ne pas parler français" | OUI | NON | OUI |
| **Représentation modalité** | Occasionnellement | Occasionnellement | Jamais Ex : « pour moi, le document qu’on lui remettait c’était une source d’information en plus, et qu’il nous montrait que ça ne lui posait pas de problème » | Occasionnellement | Souvent Ex : “s’il avait eu document, n’aurai pas forcément imprimé quel était le problème de santé” | Jamais Ex :“interprétation de ma part, peut être que le patient aurait pas remplit ainsi. Mais à mon avis il comprenait.” | Parfois |
|  |  |  |  |  |  |  |  |
| **Compréhension du sujet abordé selon le professionnel** | OUI  Ex : « l’implication des patients dans la compréhension de leurs états de santé beaucoup pour le suivi des patients à domicile » | OUI Ex :"Évaluer que les patients aient bien saisi les informations qu’on leur a donné" | NON | OUI | OUI | OUI Ex :“Est-ce que les gens comprennent bien, est-ce qu’il comprennent les termes et ce que leur état de santé est la conséquence de leur attitude” | NON  Ex :« Je vois un peu l’idée de un peu lire... » |
| **Difficultés des questions** | COMPLEXITÉ  Ex : « c’est pas difficile mais la patiente que j’ai choisi c’est la patiente parfaite donc j’ai un peu dévié » | SIMPLES | QUESTION SPÉCIFIQUE DIFFICILE | COMPLEXES  Ex : "A compris les termes au fur et à mesure, Q2 permet de mieux comprendre Q1" | COMPLEXES  Ex :"Patient vu qu'une seule fois donc confusion avec d'autres" | QUESTION SPÉCIFIQUE DIFFICILE | COMPLEXES Ex : "Evaluation à distance pas évident, parler de fréquence pas évident" |
| **Remarques** | « La première question honnêtement ça me gêne un peu « ces formulaires médicaux » je vois pas bien » | "Questions sont longues, Q3 redondante et difficile à comprendre, nécessité de comprendre l’utilité pour l’utiliser en routine" | « Je ne suis pas sûre que beaucoup de gens savent ce que c’est que la littératie en santé, je ne sais pas si ça nous parlerait beaucoup » | Pense beaucoup à un patient dans une étude et littératie lié à psychoéducation. | Première question trop anglophone : adaptation comme “est-ce que le patient peut se fier…” ou “se fait-il confiance… ?” Car ‘confident’ c’est être sûr de soi | NA | « Intéressant pour savoir comment on va accompagner le patient. Score rapide même si pas parfait peut mettre en avant si on a quelque chose à faire. » |
| **Effort réalisé** | DIFFICILE  Ex : « Ce qui est compliqué c’est pourquoi on arrive à cette réponse » | FACILE Ex : «Rapidement, parce que c’était proche dans le temps, j’ai plus mis le temps à comprendre les questions qu’à me projeter dans la réponse » | QUESTION SPÉCIFIQUE DIFFICILE | NA | FACILE Ex : "rapidement car vu le patient il n'y a pas longtemps", "problématique assez récurrente" | FACILE Ex : “Je connaissais bien le patient” | DIFFICILE  Ex :"On a l’impression qu’elle fait bien mais des fois, il y a des choses qu’elle fait pas bien et je sais pas trop comment l’évaluer" |

Continuation of the table S3

|  | **PS 8** | **PS 9** | **PS 10** | **PS 11** | **PS 12** |
| --- | --- | --- | --- | --- | --- |
| **Q1** |  |  |  |  |  |
| **« être confiant »** | CONFIANCE EN SOI | CONFIANCE EN SOI “se sentir en capacité de pouvoir le faire” | CONFIANCE EN SOI | CONFIANCE EN SOI | CONFIANCE EN SOI |
| **« formulaire médical »** | OUI  Ex: "formulaires avant hospitalisation", "formulaires sur crises suicidaires" | OUI  Ex: "Questionnaire pour les assurances (demande de prêt)"; en milieu hospitalier : "questionnaire de satisfaction", "consentement pour des examens" | OUI  Ex : "check-lists à cocher (comme bilan pré-anesthésique, formulaire de génétique)", "formulaire de consentement", "formulaire d’entrée" | OUI  Ex: “documents pour demander des prestations en lien avec son état de santé ou information en lien avec son parcours de soins hospitalisés, programmer un acte” | OUI  Ex: “demander pour ses antécédents, sur sa pathologie, des trucs héréditaires dans sa famille etc.” |
| **Situations référentes** | NON "pas de formulaire médical remis pendant hospitalisation" | OUI Une consultation, une patiente lui a demandé de l’aider à remplir un questionnaire pour son assurance | NON | NON | NON “Je me suis mise à la place” “Je l’ai imaginé qu’il est en train de me répondre” “On leur pose des questions et il répond et nous on remplit sur le formulaire” |
| **Représentation modalité** | ASSEZ CONFIANT Ex : "Je ne peux pas dire à 100% car situation de la voir remplir des formulaires ne s'est pas présentée" | PAS DU TOUT CONFIANT Ex : “Moi j’estime que si elle demande mon aide ce qu’elle ne se sent pas en capacité de le faire seule” | ASSEZ CONFIANT Ex : « Il me semble qu’il n’est pas très confiant sinon il viendrait tout seul à ses rendez-vous et il ne serait pas un peu drivé par son épouse » | PAS DU TOUT CONFIANT Ex :"Autonomie très réduite, se repose sur ses proches pour tâches basiques de la vie quotidienne. Lit mal le français." | MOYENNEMENT CONFIANT |
| **Q2** |  |  |  |  |  |
| **« demander de l'aide »** | Professionnel de santé / sur la maladie ou les soins | Proches / sur la documentation et sur la complexité des termes | Proches / questions orales / sur la documentation et sur les soins | Proches et professionnels de santé / aide à la lecture / sur la documentation | Proches et professionnels de santé / questions orales / sur la maladie |
| **« documentation remise à l'hôpital »** | Documents généraux | Documents spécifiques (relatif a la maladie, aux soins, informatif/éducatif sur sa maladie) Ex : “ça peut être une ordonnance, un résultat d’examen, un résultat de compte-rendu de prise en charge, un document d’information sur un traitement, sur une maladie, un compte-rendu opératoire, une prescription d’analyse à faire” | Documents spécifiques (relatif a la maladie, aux soins, informatif/éducatif sur sa maladie) | Documents généraux | Documents généraux / documents spécifiques (relatif à la maladie, aux soins) |
| **Situations référentes** | NON | OUI Consultation | OUI Consultation | NON | NON |
| **Représentation modalité** | OCCASIONNELLEMENT | TOUJOURS Ex : “Je suppose que tous les documents qu’on lui remet en lien avec ça, ça doit lui poser problème” | SOUVENT Ex : « Jamais, toujours, je les élimine d’office, parce que ce n’est jamais jamais et ce n’est jamais toujours » | TOUJOURS Ex : "Patient a beaucoup de mal à lire mais sa femme l’aidait beaucoup." | SOUVENT Ex : “repose des mêmes questions tout le temps par rapport à sa prise en charge” |
| **Q3** |  |  |  |  |  |
| **« difficultés à comprendre »** | Complexité de la maladie, manque de motivation | Complexité des termes, complexité de la maladie Ex : “il n’y a pas que comprendre le sens des mots, il y a vraiment ‘comment je vais pouvoir aussi me saisir de tout ça après pour ma santé’” | Complexité de la maladie, manque de motivation ex : « il y a des choses qu’il comprend mais qu’il ne veut pas comprendre, il préfère les mettre de côté » | Complexité de la maladie, complexité des documents, littératie générale | NA |
| **« état de santé »** | Maladie en elle-même | Maladie en elle-même Ex : “je comprends tout ce qui fait la santé d’une personne, à la fois ses paramètres physiques, biologiques, psycho-sociaux au sens globale” | Maladie en elle-même Ex : « sa pathologie cardiovasculaire et les mécanismes qui mènent » | Maladie en elle-même | Maladie en elle-même |
| **"difficultés à lire"** | Na | Complexité des termes Ex : “elle lisait bien, en fait, mais ça ne faisait pas sens pour elle” | Littératie générale | Complexité des termes | Lisibilité Ex : “comment s’est présenté, si c’est écrit en trop petit” |
| **Situations référentes** | NON | OUI Consultation | OUI Consultation | OUI Consultation | NA |
| **Représentation modalité** | JAMAIS | SOUVENT Ex : “J’ai pas mis toujours parce que je me suis dit ‘elle sait lire’ en fait” | PARFOIS | SOUVENT | SOUVENT Ex : “Patient qui sollicite souvent de manière générale mais pas forcément sur les documents mais sur la prise en charge.” |
|  |  |  |  |  |  |
| **Compréhension du sujet abordé selon le professionnel** | OUI Ex : "Se repérer, savoir s’orienter, gérer son autonomie dans le parcours de soins" | OUI | OUI | OUI  Ex : “Les 3 questions servent à savoir si un patient, une fois des informations données, est apte à les comprendre, se les approprier et est capable de répondre aux demandes du système de soin pour favoriser sa propre santé” | OUI Ex : "Prise en charge a besoin d’être le mieux possible donc quand patient en confiance et qu’il comprend alors il est en meilleure santé." |
| **Difficultés des questions** | NA | SIMPLES | SIMPLES | SIMPLES | FACILE Ex : “Mais peut être que je mélange tout car les questions se ressemblent plus ou moins” |
| **Remarques** | "Est-ce que le soignant peut estimer le niveau de son patient en 3 questions ?" | « Ça mérite quand même de bien connaître le patient » Questions ambiguës et restrictives | “C’est l’estimation que je fais de la compréhension de cette personne” | NA | Faire plus simple dans les questions. |
| **Effort réalisé** | FACILE | FACILE | FACILE | DIFFICILE | FACILE |

PS: Professionnel de santé or Healthcare professional, NA: Non application, NK: Not Known, Ex: Example, BPCO: Bronchopneumopathie chronique obstructive or chronic obstructive pulmonary disease
